# An ethical assessment of professional opinions on concerns, chances, and limitations of the implementation of an artificial intelligence-based technology into the geriatric patient treatment and continuity of care

**DOI:** 10.1007/s11357-024-01229-6

**Published:** 2024-06-04

**Authors:** Nina Parchmann, David Hansen, Marcin Orzechowski, Florian Steger

**Affiliations:** Institute of the History, Philosophy and Ethics of Medicine, Ulm University, Oberberghof 7, 89081 Ulm, Baden-Wuerttemberg Germany

**Keywords:** Artificial intelligence, AI-based technology, Medical ethics, Clinical decision support system, Geriatrics, Multimorbidity

## Abstract

With the introduction of an artificial intelligence-based dashboard into the clinic, the project SURGE-Ahead responds to the importance of improving perioperative geriatric patient treatment and continuity of care. The use of artificial intelligence to process and analyze data automatically, aims at an evidence-based evaluation of the patient’s health condition and recommending treatment options. However, its development and introduction raise ethical questions. To ascertain professional perspectives on the clinical use of the dashboard, we have conducted 19 semi-structured qualitative interviews with head physicians, computer scientists, jurists, and ethicists. The application of a qualitative content analysis and thematic analysis enabled the detection of main ethical concerns, chances, and limitations. These ethical considerations were categorized: changes of the patient-physician relationship and the current social reality are expected, causing de-skilling and an active participation of the artificial intelligence. The interviewees anticipated a redistribution of human resources, time, knowledge, and experiences as well as expenses and financing. Concerns of privacy, accuracy, transparency, and explainability were stated, and an insufficient data basis, an intensifying of existing inequalities and systematic discrimination considering a fair access emphasized. Concluding, the patient-physician relationship, social reality, redistribution of resources, fair access, as well as data-related aspects of the artificial intelligence-based system could conflict with the ethical principles of autonomy, non-maleficence, beneficence, and social justice. To respond to these ethical concerns, a responsible use of the dashboard and a critical verification of therapy suggestions is mandatory, and the application limited by questions at the end of life and taking life-changing decisions.

## Introduction

Research and the development of medicine has contributed to generally extend the duration of peoples’ lives and simultaneously to a rising number of older people [1]. This development of a changed distribution of ages and continuously growing number of the older population creates new global challenges, especially regarding the distribution of resources in medical care. Since the rate of multimorbidity drastically rises at the age of 65 [2], the increasing number of older people leads to a rising number of people suffering from multimorbidity. Patients with multiple illnesses are in need of an individualized and patient-centered treatment. They tend to be more often admitted and have longer stays at the hospital and a higher rate of polypharmacy than non-multimorbidity patients [3]. Considering these factors, an increasing number of multimorbidity patients require a rising number of resources. The number of hospital beds in Germany will decrease by about 30 percent until 2030 [4], while the number of patients and the demand of an individualized treatment is drastically rising. Therefore, new medical solutions need to arise to assure a fair and full coverage of medical care.

The development of a dashboard within the project “Supporting SURgery with GEriatric Co-Management and AI” (SURGE-Ahead) responds to the increasing number of geriatric patients and simultaneously decreasing resources in the geriatric medicine, primary in the surgery wards. This Artificial Intelligence-based device will automatically process and analyze patient data to generate an evidence-based evaluation of the health condition as well as recommendations for therapy. The introduction of this diagnostic and therapy application supports physicians and the nursing staff to enable an improvement of individualized treatment and continuity of care.

Studies have shown that the introduction of artificial intelligence (AI) into the clinical care makes a positive impact in various fields in medicine and offers new possibilities: diagnoses become more accurate, individual treatment speeds up [5], and the general quality of patient care improves [6]. Additionally, the physicians’ workload might overall be reduced, the medical performance enhanced, and simultaneously stress decreased [6]. AI has been proven its feasibility of processing complex data to detect diagnostic findings and make health-related predictions [7]. Considering these results, the implementation of the AI-based dashboard into the clinic can be expected to improve individual geriatric treatment and perioperative care.

Despite the variety of possibilities and improvements that arise with the introduction of AI into medicine, a development and introduction of an AI-based dashboard for medical evaluation and treatment recommendations in the geriatric medical care lead to diverse ethical questions. Hence, we aimed to determine subjective opinions on medical ethical risk and benefits of the implementation of an AI-based support-system in the geriatric medical care through the conduction of qualitative interviews with experts of geriatrics, computer science, law, and ethics.

## Methods

In order to gain professional opinions on chances, limitations, and ethical concerns on using the dashboard in the clinical practice, we have conducted semi-structured qualitative expert interviews. This method allows exploring subjective opinions of the interview partners and simultaneously provides flexibility in the conduction of the interviews. Thus, the interviewees can emphasize and expand on viewpoints that are important from their professional perspective. Simultaneously, this method enlarges the option of the interviewer to adapt the interview structure to the participant’s answers, for example through the possibility of asking questions spontaneously [8].

To ascertain multiple professional perspectives, experts in four specialist fields were included: head physicians, computer scientist, jurists, and ethicists. To limit the range of specialists, we used a narrow definition of experts: potential participants we contacted were supposed to have specific and technical knowledge that targets onto a professional field into which it is potentially being applied into [9]. Additionally, certain criteria needed to be filled before interview requests were sent via e-mail: the head physicians should work in geriatrics; computer scientists should be specialized in AI and its medical application; jurists should be specialized in AI, digitalization, and protection of private data; and ethicists should have expertise in AI, digitalization, or medicine. An intensive web search as well as snowball samplings were conducted to gain criteria-fitting participants. Finally, the interviews were conducted with 19 experts from Germany and Austria: five head physicians, five jurists, five ethicists, and four computer scientists.

In advance of the interviews, the participants were informed in writing and verbally about the background, funding, and aims of the project. Furthermore, they were educated about the process of the interview, the further processing of the interview material, protection of their privacy, and the voluntary participation in the interview. This included the option of withdrawal until the anonymization of the transcripts without any consequences. All interviewees have given consent for the participation in the interview.

The questionnaire was divided into five main categories: general ethical position of the participant on the topic of the research, issues regarding patient autonomy and digital competences, chances and risks of the dashboard’s introduction into clinical practice, and considerations on social justice. The interviews were concluded with the option to add or highlight additional or particular aspects. The questionnaire was applied in interviews with all expert groups. Hereby the comparability of the interviews could be maintained. The interviews were conducted in German between September and December 2023 via the Zoom Video Communication platform to allow adaptation to each others schedule and to be placed independent. The interviews were digitally recorded, transcribed by an external company and subsequently anonymized. The transcripts of the interviews were analyzed in German, and representative quotes from the interviews were translated from German into English.

The transcripts of the interviews were analyzed following the qualitative content analysis [10] and thematic analysis [11, 12] to enable the detection of the main thematic categories in the interviews. Therefore, we first implemented an inductive formation of categories and sub-categories based on the interviewees’ statements. Hereafter, a deductive analysis according to main five categories, into which the interview questions were structured, was conducted. The content of the interviews was coded according to the established categories, and corresponding quotes and contents were categorized. Interviews were initially analyzed separately within expert groups before comparing, combining, and stating similarities as well as differences across the groups. After individually conducting the qualitative content analysis, findings were discussed. Four researchers (N.P., D.H., M.O., F.S.) with technical expertise of medicine, medical ethics, legal studies, and philosophy discussed thematic categories, revised, and concluded results.

## Results

Main ethical concerns, chances, and limitations regarding the introduction of an AI-based dashboard into clinical perioperative geriatric care that were repeatedly expressed throughout the interviews could be classified into five categories: (i) patient-physician relationship, (ii) social reality, (iii) resources, (iv) fair access, and (v) data-related aspects of the AI-based system.

### Patient-physician relationship

According to the participants of the interview, main changes that are expected in the patient-physician relationship will develop in the patient-physician communication as well as the role of the physician, patient, and the AI-based dashboard. Our interview partners stated that distrust in AI would cause distrust towards the physician, while trusts could strengthen the patient-physician relationship.

**Fig. 1:**
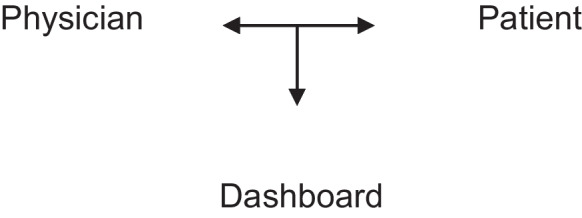
Representation of the balanced relationship model

Different new models of a triangular patient-AI-physician relationship were expected. One possibility would be a balanced relationship (Fig. 1) of the patient and physician, in which the physician’s role would turn from a sole decision-maker into an advisor. Here, the physician’s new role was described as a sole communication service provider, whereas the AI would assume the role of an expert. Thereby, the physician would lose his current role of being a medical expert and decision-maker with therapeutic freedom. This could result in patients becoming uncertain about whom to trust and starting to question the physician’s authority. One expert suggested a development of a shared decision-making of all three parties involved, which would be based on a levelled relation. However, another expert reasoned physicians would tend to rather keep the support of the dashboard in the background.

**Fig. 2:**
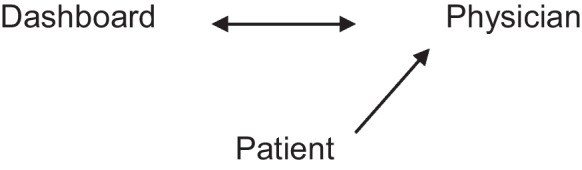
Representation of the equal relationship model

Another model described by the interviewees involved an equal relation (Fig. 2) of the physician and the dashboard. The internal communication would be processed in a negotiation process between the physician and the dashboard, while the patient would assume a sole role of a “data source.” The communication was expected to become sterile and alienated and with it, to lose the therapeutic effect. Especially this development was considered to aggravate the communication in the geriatric medicine considering patients desiring interaction and verbal communication.

**Fig. 3:**
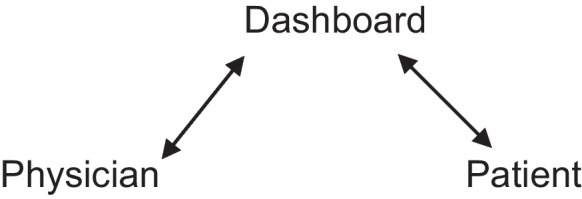
Representation of the AI-mediated relationship model

The third model described by the interviewees presents AI as a mediator and leader of the communication (Fig. 3). The dashboard was expected to function as a transmitter of inconvenient truths and responsible participant, which one physician described as follows:

It would probably make my life a lot easier. Because in principle, one would hand over a lot of responsibility. One would say “Actually, the Artificial Intelligence has told me that we should do his [patient] surgery,” and then afterwards, if somebody comes “You should not have done the surgery”, then I would say, “Yes, but the system has said something different*.* (head physician)

### Social reality

The interview partners anticipated changes in the social reality that would mainly involve establishing AI as a new participant, the loss of professional skills, as well as changes in social practices.

AI was predicted to become a new participant due to its active influencing or undertaking decisions. Several experts described AI as unconscionable, emotionless, and devoid of empathy.

(…) AI is inherently conservative (…) (ethicist)

The technology was anthropomorphically described as “colleague AI” and “very young colleague”. It would neither be possible to discuss nor argue with it; however, it would behave similar to humans in its verbal and written expressions.

A further challenge would be de-skilling. Due to the increasing takeover of work stages by the technical system, physicians would no longer need to process information by themselves. As a result, physicians could lose medical competences. They would become unable or, at least, less able to process and evaluate information that is necessary for diagnostic and therapeutic decisions; they would become dependent on the technology and develop a risk of over-reliance.

In addition, interviewees stated that the technical implementation would change the conceptualization of illnesses, age, and suffering.

### Resources

According to the participants of the interviews, the introduction of the AI-based dashboard could affect different kinds of resources: human resources, time, knowledge, and experiences, as well as expenses and financing. Changes in the development could establish new chances but simultaneously result into ethical concerns and limitations of the technology.

#### Human resources

AI is expected to reduce the workload in the clinic and support healthcare professionals with complex expertise. The interviewees mainly anticipated essential support in repetitive tasks, including preselection of findings and the organization of polypharmacy. The device was considered as being able to assist the medical team with specialist knowledge and to balance out absent geriatric specialists. Nevertheless, interviewees believed that the introduction of the dashboard would require a changed composition of knowledge and specialization. An increased number of trained staff would be necessary to enter and edit patients’ data, educate patients, and monitor technical devises.

If I still have to use the AI program with about 30 questions that I have to work through, I will practically have more work than before. (head physician)

Computer scientists were considered to become indispensable to explain the decision-making process of the AI to the medical staff and for the overall technical support.

#### Time

The implementation of the dashboard would enable a quick information access and diagnostic analysis as well as an acceleration of several processes, such as an earlier detection of risk patients, disease outbreaks, deterioration of health, and detection of new phases of diseases like dementia. However, the additional administration of data and evaluation of generated suggestions, time-consuming consent discussions, and an enduring adaptation phase would cause additional time consumption. Moreover, regular employee trainings on digitalization, the use, access, data processing, and the approach of dealing with results would need to be implemented. Considering hectic daily routines, physicians were rather expected to blindly rely on and accept the suggestion, especially in consideration of the high workload and previous experiences of the dashboard’s opinion as valid.

What are the chances that in a hectic day-to-day clinic routine, the physician will ask in each case, „Alright, this is an interesting suggestion. I have to think if it really is a good one.” Or are you more likely to say, “Well, the system will be right.”. That it somehow develops a momentum. (ethicist)

Despite a quicker, more efficient, and precise quantitative data processing and analysis by computers, the interviewees highlighted humans as being better in qualitative data analysis.

Actually, I know what the outcome will be, but I have to do it, because otherwise I cannot discharge the patient. That would rather steal resources. I think even with a well-functioning system; it will remain a zero-sum game. (head physician)

While some interviewees expected physicians to invest the time savings into interactions and dialogs with patients, other interlocutors emphasized the systematic pressure and the use of the saved time into further patient treatment. Generally, the interview partners stated the dashboard would not be a time saving tool in geriatrics but would rather cause a redistribution of workload and time.

#### Knowledge and experiences

The interviewees described the dashboard to become a source of broad knowledge that additionally would consider the current state of medical research. The complex knowledge was considered to be a major decision support in hospital admission and discharge. Additionally, the correlation of specific data would enable accurate and precise results. Nevertheless, the interlocutors questioned AI to be able to integrate subjective experiences. Although the device’s performance would function independent of human factors like fatigue, the physician’s state of knowledge, and working hours, AI should not be used in decision-makings, in which human factors and intuition are indispensable, such as the end-of-life decisions, palliative medicine, and cases of severe dementia. Interviewees emphasized technical device to be unable to detect the assumed will of the patient that would only be possible to be uncovered in human interaction and through conversations.

Furthermore, interviewees highlighted the risk of misuse. Physicians were expected to use the system not in a way that is recommended.

What people do, they turn off patient’s drugs and see how the risk potential changes. But I know this algorithm is not trained for it. It is called counterfactual reasoning. (…) And here I say, I cannot use it for that purpose. (…) And that is a huge problem. But that is not the mistake of AI, that it does not work or that it cannot be used that way, but the user is using it wrongly*.* (computer scientist)

#### Expenses and Financing

The introduction of an AI-based technology was considered to be a high investment that raises main concerns in the financing of the dashboard. Economic constraints and factors might default into the technical device. Conflicts of interest and influences of sponsors could affect the development and decision-making performance of the tool.

Theoretically, if we now take the orthopedic field, there might be a prosthetic company behind it, so that every suggestion from the dashboard results in a new hip prosthesis*.* (head physician)

The dimension of the financial influence was expected to depend on the financial funding from either public, scientific, or commercial sources.

### Fair access

As a basis for fair access, the interviewees expressed the requirement of training the AI on a cohort that would not lead to discrimination. Nevertheless, the interviewees criticized existing errors in the data and the impossibility to clean the dashboard from false and discriminating data. Therefore, interlocutors expressed the concern that existing inequalities would be intensified in further training and development of the base algorithm.

Any inequalities that exist are usually found again in the data. And therefore usually, not necessarily, but often, continued in the suggestions*.* (ethicist)

The number of false data would increase with increasing data input. The interviewees criticized insufficient data basis of certain diseases. To prevent false diagnosis and therapies, they advised to only apply the dashboard on patient groups that were represented in the trained cohort. The dashboard was described as an objective support system. Nevertheless, interviewees stated that patients would prefer a subjective assessment of the physician. Especially in geriatric medicine, the individual factor was described to be significant when evaluating decisions of transferring patients into care homes or rehabilitation. The interviewees doubted that the dashboard’s sole orientation towards data could identify the daily condition of patients, especially when considering dementia as a particular individual illness. Furthermore, one expert questioned the consideration of individual target parameters, like the receipt of mobility. Social determinations, including health-related behavior, biographic backgrounds, and the social status would not be recorded despite the correlation of demography and income with diseases. The interview partners pointed out that geriatric medicine, as a field of a vulnerable patient group, involves the risk of ageism. Defaults in the technical system would possibly integrate age limits for certain therapy options. Additionally, the interviewees stated the possibility of the software-engineering and political guided influence on the software. A systematic discrimination could be implemented into the data set to discriminate against certain age or physical disabilities.

### Data-related aspects of the AI-based system

Interviewees raised main concerns regarding to data-related aspect of the AI-based technology including privacy, accuracy, accountability, transparency, and explainability.

#### Privacy

The reduction of privacy was expected. The patients’ loss of the ownership of their private data and further use for research purposes was questioned, and the unawareness of patient’s data use was described as an invasion of the privacy rights. Moreover, concerns were raised that anonymized data sets would not longer be governed by specific liability regulations and about the risk of individual-related data to be re-identification from the allegedly anonymized data with new technology in the future. Interviewees doubted that hospital staff will be sufficiently trained in data protection and stated their concerns about cyber security.

The more you rely on a software, the more you need security against unauthorized data access (…) (jurist)

However, one expert emphasized data protection and data security would not only restrict the number of available data but additionally the accuracy of diagnosis and therapy decisions.

#### Accuracy

The interviewed experts emphasized the possibility for a quick diagnosis and an accurate therapy suggestion that based on a large number of data. Others doubted its accuracy would depend on the data selection and raised concerns about the completeness of data.

Is the quantity of training data sufficient for the model? Some result is always generated (…) (computer scientist)

A further concern was the currency of the data and the critic of false suggestions of treatments caused by obsolete data. A jurist estimated AI to be reliable as long as circumstances remain stable. New diseases or types of treatments that have not been the basis of training for the system would not be able to be considered by the technology.

#### Transparency

Our interviewees expressed the necessity to disclose information of the data processing, especially regarding the data source, data flow, and whether data is transferred to a third party.

Does the data stay in the clinic or is it transferred to the producer of these things? I was involved in [task and location anonymized]*.* And there we faced the problem that the data was too complex to calculate (…), so we had to send it to a data center in [place anonymized]. This is a whole other dimension of data processing. Such things do need to be clear. (ethicist)

Considering legal regulations, patients would need to be informed about the use of AI and its meaning, whereas the internal use of data would not require any data protection approval. A major concern included the prospective use of the dashboard. A jurist emphasized that the dashboard as a tool being developed and introduced within the scientific scope, would cause neither transparency issues nor dilemmas concerning the data protection law. However, after its development, major difficulties could arise when commercializing the product if developers do not disclose their model. The certified medical product would be expensive, and usually neither scientific nor non-profit institution or medical expert associations would be able to fund it. Only commercial providers could cover the expenses. Therefore, transparency and a reduction of existing inequalities could only be assured when the dashboard was funded by a solidary system.

#### Explainability

A general concern of the interviewees was that AI could be considered as a “black box” and lead to the incomprehensibility of its decisions. The basis of the data processing would not be explainable, and the data source was described as impenetrable. The input and output would be comprehensible, but the inner mechanism difficult to understand, even for scientists.

[Artificial Intelligence is a] High complex decision-making, that is not, or not well explainable, even if we have the entire decision path. Because it is a whole chain of connections and links. (computer scientist)

Scientists could just hope that they have tested the system often enough, and the output and input of the system seem plausible. Furthermore, the system was described as epistemic opacity. Neither the patient nor the physician would understand how the AI functions. This unexplainability would cause insecurity. Therefore, interviewees expressed the importance to include an audit trail into the system to enable a pursue of changes in data and ensure auditability. Concerning the auditability of the data source, a computer scientist was concerned about the feasibility of the anonymization or pseudonymization of patient data.

## Discussion

The main ethical concerns, chances, and limitations that were expressed by the participants of the interviews are further discussed considering the four principles of Beauchamp and Childress: autonomy, non-maleficence, beneficence, and social justice [13] (Table 1).

**Table 1:** Representation of the ethical concerns of interviewed experts and the corresponding ethical principles that could be jeopardized after introducing an AI-based dashboard in the geriatric medical care

Autonomy Non-maleficence Beneficence	**Patient-physician relationship**	Balanced: physician and patient; AI as an expert Equal: physician and AI; patient as a data source AI as mediator
Autonomy Beneficence	**Social reality**	AI as new a participant Re- and upskilling
Autonomy Non-maleficence Beneficence Social justice	**Resources**	Redistribution of the following: Human resources Time Knowledge and experience Expenses and financing
Non-maleficence Beneficence Social justice	**Fair access**	Insufficient data basis Intensifying of existing inequalities Systematic discrimination Consideration of the patient’s daily condition
Autonomy Non-maleficence Beneficence Social justice	**Data-related aspects** **of the AI-based system**	Privacy Accuracy Transparency Explainability

### Patient-physician relationship

The first model described by the interviewees is a balanced relationship between the physician as an advisor to the patient and the AI that participates as an expert. Hereby, the physician requires broad clinical and technical understanding to be able to sufficiently advise [14]. Nevertheless, due to the possible major lacks in the explainability of the AI and therefore the physicians’ inability to fully explain and understand the output of the technology, patients are likely to question the trustworthiness of physicians [15]. This triangular relationship might jeopardize the principles of autonomy and non-maleficence. If physicians do not fully understand the basis and decision-path of the dashboard, they are unable to accurately advise the patient and simultaneously risk incorrect suggestions. Furthermore, considering that the physician’s suggestion is not based on a full understanding of the AI’s decision path, patients’ autonomous decision taking can be questioned as well as the maintenance of the physician’s therapeutic freedom to act autonomously.

The second model was described as an equal relationship between the physician and the dashboard, while the patient would be reduced to a data source. The development from a caring to a sterile and solely data-based communication would neglect human-based factors in medical decision-making. Previous research has shown that the patient communication and emphatic skills require human competence, which however cannot be replaced by a technology [16]. Hereby, the principle of beneficence might be harmed. Especially in the geriatric medicine, where the patient might be situated in a long-term patient-physician relationship based on trust and human interaction, the new AI-based participant could cause mistrust in the physician’s suggestions. This mistrust might lead to a reduction in the therapeutic effect and to the patient’s feeling of not being medically and humanely cared for.

The third model described the participation of the AI-based device as a mediator. The mediating effect of a technology has already been proven by researcher analyzing the experience of smartwatches [17]. This development of the AI-based technology being recognized as a transmitter and responsible participant raises strong concerns about the ethical principles of non-maleficence and beneficence. The assumption of not being responsible for the consequences of medical treatment decisions reduces precise and accurate treatment considerations. The blind trust in the dashboard’s decision increases and with it, the risk of medical errors.

#### Social reality

The interviewed experts estimated major changes in the social reality after introducing an AI-based dashboard into the clinic. The AI would appear as a new participant and “colleague” that lacks empathic and communicative skills. The working collaboration with an “AI-colleague” is perceived as less meaningful, less motivational, as well as less satisfying when compared to a “human-colleague” [18]. The perception of the AI to have human skills and expressions might contribute the approach of minimizing estranging experiences of a new opaque technology. The contradiction of the AI to be perceived as colleague that however lack of human skills might negatively conflict with the practice of medical profession. Therefore, the satisfaction and meaningfulness of the medical profession might decrease and with it the effort of an individual and patient-centered treatment. This development could conflict with the ethical principle of autonomy and beneficence.

Moreover, interviewees assumed a de-skilling of the professionals that results from the reliance on medical suggestions. The introduction of a dashboard that takes over the data processing and generating of suggestions might lead to a reduction of the critical and complex medical thinking. The changed clinical routine might lead to medical de-skilling of communication skills, clinical knowledge, and the recognition of the patient as an individual [19]. Nevertheless, the implementation of technical education programs is expected to simultaneously lead to a re- and upskilling process: New technical skills and competences are likely to develop and enhance an alteration of medical skills. These new skills enhance the technical understanding of the AI-based device which leads to the benefits of the patients.

#### Resources

##### Human resources

Interviewed experts assumed a change in the distribution of professionals and workload. An AI-based device functions efficiently and processes data rapidly [20]. Therefore, the workload of physicians can be reduced by supporting the evaluation of medical and patient data. However, the introduction of the technical device requires a close cooperation between physicians and computer scientists [21, 22]. Professionals in the medical field might be reduced, but simultaneously the number of technical staff and computer scientists will have to increase. Rather than a reduction, a redistribution of professionals from different departments can be expected that is composed of an increased number of technical professionals as computer scientists and a decreasing number of medical professionals. However, the massive shortage of computer scientists in Germany [23], that is necessary for a sufficient technical and medical support, could not be ensured. Considering that the use of the dashboard, and therefore the patient’s treatment would depend on the medical and technical understanding of the physician as well as the efficiency of the new cooperation, the principles of non-maleficence as well as beneficence could be threatened.

##### Time

Participants of the interviews assumed a redistribution of time and workload when introducing the AI-based dashboard into the clinic. A support of the application would provide a quick data access, diagnosis and findings of therapy options. While some interviewees described the saved time to be invested in patient-physician interactions others however assumed that rather more patients would be treated to keep up with the increasing workload. The assumption of rather investing the saved time for economic benefits and treating more patients is likewise described by previous research [15]. Blind trust into the technical device as an option for coping with stress and saving time can be expected to become standard. This development jeopardizes ethical principles of non-maleficence and beneficence. In particular geriatric patients require more time to fully open up in a possibly overwhelming clinical environment, and the consideration of multimorbidity and cognitive impairment requires additional time sources. A decreased time in the patient-physician conversation increases the chances of overseeing symptoms, patients not mentioning symptoms that subsequently increases risks of incorrect diagnosis and therapies.

##### Knowledge and experiences

Interviewees described the dashboard as a source of broad knowledge that provides accurate and precise results, functions independent of human factors but simultaneously is at risk of misuse. The use of the technical device is related to the physician’s individual sense of responsibility, subjective understanding of liability and trust in the device. Previous research has shown that physicians will follow the medical suggestions if the device’s suggestions are in line with their own medical judgement [24]. An AI-based technical device is proved to generally enable individual diagnosis and therapy, an earlier detection of diseases, and an improved treatment leading to a better quality of life [25]. It enables a similar knowledge basis for physicians regardless of their professional experience, better medication adjustments, in particular polypharmacy, and simultaneously lowers the risk of a potential harmful drug combinations [26]. Considering the ethical principles, chances as well as concerns can be identified. The application of the AI-based device might reduce the risk of wrong therapy and therefore support the principle of non-maleficence. However, despite a good intention of better and quicker treatment of patients, the risk of wrong application might lead to unintended harm of patient’s well-being. Furthermore, errors in the clinical routine based on human factors will remain, but distribute: due to fatigue, convenience, or exhaustion, physicians might not question dashboard’s suggestions and blindly trust on its liability. Therefore, the principles of non-maleficence and beneficence might be jeopardized. Considering the principle of social justice, the similar broad source of knowledge could allow patients to be treated independently of their physician’s knowledge and medical experience. Nevertheless, the individual use of the technical tool will remain a significant factor. When verifying generated suggestions, the classification and evaluation will in most cases remain dependent on physicians’ professional medical knowledge and experience.

##### Expenses and financing

Participants in the interviews expressed concerns about financial interests and conflicts of interest being based on commercial funding and sponsorship. These worries are consistent with current research. The development of an AI-based decision-making device is described to be susceptible to manipulation and guided influence. Financial and commercial conflicts of interest are likely to arise from the device’s involvement of parties as economy, developers, and clinicians, and are not necessarily responding to the favor of patient’s interests [24]. These impacts are conflicting with all four ethical principles [13]. Considering an AI-generated suggestion that is based on, for example, commercial interests, an autonomous decision cannot be assured. Furthermore, the principles of non-maleficence and beneficence are harmed through putting patients’ well-being on risk favoring, for example, commercial interests and the patient’s care being dependent on embedded interests. In addition, social justice cannot be assured considering a differentiation of treatment that depends on biases in the algorithm.

#### Fair access

When implementing an AI-based dashboard into the clinic, interviewees expressed concerns about an insufficient data basis, the intensifying of existing inequalities, the possibility of systematic discrimination, as well as the impossibility of the consideration of the patient’s individual daily condition. Research shows that accuracy depends on various factors that are necessary to be integrated into the training cohort [27].

A main data source for the training of the AI-based dashboard is existing patient data, which, however, could be erroneous [28]. Considering that the group of geriatric patients has a high number of cognitive impairments, the reliability of stated data during the anamnesis cannot be assured [29]. Over- or understating symptoms might lead to misjudging the importance and relevance of symptoms, resulting in incorrect data administration. Often these errors are detected by physicians’ experiences, unlike an algorithm that is based on data input [30]. These errors will be integrated into the data set and become a part of the basis of the learning algorithm. Another aspect is the unconscious integration of biases and discriminations into the data that is influenced by personal imprinting. Intentional and systematic, for example, political biases can be installed for funding reasons, influencing unfair decision-making, which users do not necessarily recognize [31, 32]. Considering these concerns, the principles of non-maleficence and beneficence are jeopardized, if the patient’s medical care and well-being is being put on risk by incorrect suggestions that are based on an incorrect or insufficient data basis. Additionally, a systematic discrimination would counteract the principle of social justice.

#### Data-related aspects of the ai-based system

##### Privacy

Respondents expressed concerns about unauthorized data access and use, a possible re-identification of private data and cyber security. These concerns correspond to results of scientific research. Currently, established privacy-preservation methods are described as being unable to fully ensure patients’ privacy [33]. Existing tools with higher chances of protecting privacy are currently only applicable in research purposes [34]. Provided that personal data of the patient remains in the clinic, a re-identification and an allocation to the patients’ data remains feasible given the use of pseudonymization [35]. Furthermore, researchers describe that the introduction of AI algorithms raises major concerns about privacy attacks to collect sensitive patient data [36]. Considering the possibility for an unauthorized data access, security gaps, and therefore the theft and misuse of patient’s data, the ethical principles of autonomy and non-maleficence could be harmed.

##### Accuracy

Interview partners emphasized chances of accurate, individualized therapy suggestions that result from the large data basis. However, other interviewees raised concerns about the incomplete data basis that could cause false medical suggestions. Previous research has shown that the accuracy of the treatment suggestion depends on the patient group that the algorithm is trained on. Therefore, suggestions for patient groups, which were not included into the data, will lack in accuracy [37]. In contrast, another research has shown that despite missing data and parameters of certain patient groups, algorithm-generated suggestions may however be accurate for a high number of patients [38]. In consideration of both findings, the results support the indication that many diagnosis and therapy suggestions may indeed be accurate; however, correct suggestions cannot be equally assured. Considering that underrepresented patient groups face a higher risk to receive incorrect suggestions than the well-represented patient group and therefore higher risks of false treatment [39, 40], the principles of non-maleficence, beneficence, as well as social justice might be jeopardized. Nevertheless, considering the possibility of individualized and accurate medical suggestions for patient groups that were well represented in the training, the principle of beneficence would strongly be supported.

##### Transparency

Interviewed experts emphasized the necessity of the disclosure of data processing and the transfer of data to the third parties. One expert stated the concern that transparency could not be assured after the dashboard is commercialized. Similar concerns are represented in previous research. A transparent communication with patients about the data use is described as necessary [41]. Furthermore, significant concerns about the access, control, and use of data by for-profit parties are stated when commercializing healthcare AI [41]. To ensure the ethical principle of autonomy, it is necessary to inform patients about the extend, to which the dashboard influences medical decision-making. Additionally, the commercial use of the AI-based dashboard could raise serious concerns about the unauthorized use of patient’s data, which might jeopardize the principles of non-maleficence.

##### Explainability

Interviewees expressed concerns about the incomprehensibility of AI-generated decisions due to the black box model, as well as the explainable data processing. Researchers describe the input and output of the machine learning model within a black box to be comprehensible, while the inner mechanism and detailed process of the decision-making remain unexplainable; even for data scientists and system developers [42]. Hereby, neither the physician nor the patients are granted full transparency. These prerequisites of an AI-based decision-support system jeopardize ethical principle on different levels. Considering that the suggestion of the technical device does not correspond to the physician’s opinion, the option to detect the source of disagreement is highly important to ensure the principle of autonomy, non-maleficence, and beneficence. A full understanding of the decision-making process is necessary to ensure autonomous decision and the participation of the patient and further to prevent false treatments that are based on an incorrect data base and avoidance of unnecessary risks to the patient.

In conclusion, the research findings have shown that the development and introduction of the AI-based dashboard into the clinical routine raise various ethical questions in the geriatric medical care.

The introduction of the dashboard provides new chances in medicine: the dashboard would generally facilitate a quick access to a large medical knowledge and evidence-based data, regardless of the individual medical experience and education of the physician. It could enable precise individualized diagnosis and treatment suggestions and reduce medical mistakes that are based on a lack of geriatric knowledge. Additionally, the dashboard could potentially improve medical treatment and simultaneously respond to geriatric-specific difficulties as polypharmacy and multimorbidity.

Nevertheless, the development and introduction of an AI-based system raise ethical concerns regarding the patient-physician relationship, social reality, resources, fair access, as well as data-related aspects of the AI-based system. Different models of the patient-physician relationship are expected to develop which could harm the ethical principles of autonomy, non-maleficence, and beneficence. The appearance of AI as a new participant and the re- and upskilling of professionals, as a result of changes in the social reality, could conflict with the principles of autonomy and beneficence. Various resources, such as human resources, time, knowledge and experiences, as well as expenses and financing could risk the autonomy, non-maleficence, beneficence, and social justice. Furthermore, the introduction of an AI-based dashboard raises concerns about the fair access, specifically of an insufficient data basis, the intensifying of existing inequalities, systematic discrimination, as well as the consideration of the patient’s individual daily condition. Hereby, the ethical principles of non-maleficence, beneficence, and social justice could be jeopardized. Other major concerns established in the data-related aspects of the AI-based system: privacy, accuracy, transparency, and explainability could threaten the autonomy, non-maleficence, beneficence, as well as social justice.

Considering previous findings to respond to the ethical concerns, a responsible use of an AI-based dashboard is mandatory, especially in the geriatric field: the dashboard should be used within a shared decision-making process where the patient remains in the center of communication. Within the entire application, it is necessary that the AI-based device remains a support system. The use of the dashboard requires an individual review of each suggestion to form an own medical assessment as well as a responsible use despite economic constraints and hectic clinical routines. Before and after the introduction of the dashboard into the clinic, we strongly recommend a technical education about its function, scope of application, and decision-making process of the dashboard which should additionally involve a continuous cooperation with computer scientists. Hereby, users will gain awareness of the dashboard’s limitations and the correct use to prevent potential harmful misuse. To prevent conflicts of interests, which could severely influence the patient’s treatment, a commercialization of dashboard should be avoided and instead be funded by non-profit institutions or medical expert associations. To increase the accuracy of the AI-generated suggestions and reduce discrimination, we consider an integration of a sufficient variety of patient data, including minorities as crucial. The use of the dashboard requires transparency. Patients need to be informed about its usage, its broad functionality, and its range of applications. Due to major security issues and lack of privacy, patients should have the opportunity to either decline or approve the use and integration of their private patient data. Despite the wide range of technical possibilities, a limited application of the device is ethically supported. Major limitations of the dashboard’s application include medical considerations, decisions, and suggestions that are closely related to human values, such as questions at the end of life, decisions of palliative care, and life-changing decisions.

The research is limited by several conditions: the findings of this research are based on a conduction and qualitative analysis of expert interviews that include subjective opinions of the participants from Germany and Austria. Therefore, these findings do not allow a generalization in global perspectives. The number of 19 interviewees is considered as relatively high within the scope of qualitative analysis, and the findings could possibly have been detected with a lower number of participants. In addition, the expert interviews were conducted when the dashboard was not fully developed. Therefore, the participants were not provided with detailed information about this specific AI model as a basis of their assessment. Furthermore, the participating expert groups did not necessarily have professional medical insights, experience, or detailed knowledge of clinical routines; consequently, some experts based their statements on their own personal medical experience.

## Data Availability

The original contributions presented in the research are included in this paper. The corresponding author can be contacted for further inquires.
